# Things Doing and to Be Done

**Published:** 1919-02-22

**Authors:** 


### IERNE'S CRITICISMS AND APOLOGIA.

### Things Doing and to be Done.

A FRIENDLY correspondent has been good enough to send me a letter of criticism, which, I regret to observe, is marked "not for publication." From this I gather that my words have been as goads, and that my criticisms have been found in some quarters unkind, not to say unfair. I heartily thank my correspondent. Nothing appeals to me half so strongly as frank outspokenness. There is, however, lacking from this letter one very important item, being a quotation of the offensive words. A century and a half ago one John Wilkes, a newspaper man by the way, was arrested and imprisoned on "general charges." Such warrants were found to be illegal, and now delinquents are entitled to a precise specification of their alleged shortcomings. May I suggest that it is not too late to remedy the defect?

I gather, then, that in a general way I have wounded the feelings of many excellent hospital matrons for one thing, and that for another I have been urging the College Executive to accomplish in a few days tasks which really require months to complete. I propose here and now to offer my apologia, but before doing so may I say that it is hardly necessary to tell a trained nurse that sore feelings are not always harmful. What of a blister? I am a confirmed believer in the value of criticism, and I speak from personal experience. The most unwholesome atmosphere in the world is that produced in a mutual admiration society.

May I ask two favours of my readers? One is to accredit me with purity of motive. My judgment may be warped, but I am void of malice. The other is that they will endeavour to see themselves as others see them. There are people whose vision is oblique, who see men as trees walking. Even their impressions must be taken account of. When I paint a picture I try to convey what appears to at least one onlooker, and may I add that even caricaturists have their mission.

Let me begin with the College. Foolishly or not I am really concerned about the College. It is young—scarcely past its babyhood—and it is growing day by day and month by month, so that in its early days it is encouragingly robust. Without exaggeration a vast deal may be said in its praise. Praise, however, is not my mission. Please bear this in mind. I am in quest of the weak places. A chain is only as strong as its weakest link, and my business is to find that link. If I am successful and get the weak place made strong, I venture to suggest that any little pain caused by the operation is justified.

*I hold the belief, first, that the College Executive moves too slowly, and secondly, that it contains an undue preponderance of hospital matrons.*

This statement may be wholly unjustified. Nevertheless it is an honest and a strong conviction, and I propose to show why I think as I do. I court reply, and if my statement is a libel I shall be the first to ask that it may be burnt by the common hangman.

Now, owing mainly to the war, the British nursing world is in a condition very closely approximating to chaos. Its members are without organisation or cohesion, and never before in our history were organisation and cohesion so urgently necessary. If nursing is to take its proper place in the great work of Reconstruction the present condition of affairs must be revolutionised. I am, indeed obsessed with the immensity of the various tasks which confront the College, and it is likewise apparent that many of them will not suffer delay. They simply cannot be postponed to a convenient season. Take, for example, the question of nurses' hours of work and nurses' pay. This is going to be settled in the early future either by the College in a dignified and professional way, or else it is going to be settled by a Trades Union. And, I tell you, my masters, that the Trades Union, if it takes the matter in hand, will find a short cut to the ascertaining and laying down of what shall consist a maximum working day, and to fixing a minimum scale of remuneration. I am not in a panic. Within the next fortnight in Dublin there is to be held a public meeting with the object of enrolling nurses in a Trades Union. Everybody knows that the economic conditions of nursing in Ireland are scandalous. The College has been established in Ireland for a very considerable time, and the nurse has waited in vain for succour. Hence Trades Unionism. To-morrow the same story will be told in Great Britain.

Then there is the coming Registration Act. Do not make the mistake of over-estimating its potential benefits. Much has been heard of State Registration in America, but it is well known that it has failed to realise the dream. In some vague way it was hoped that State Registration would confer a monopoly. It has not done so, and now an effort is being made to promote a law for licensing all persons tending the sick for gain, including the "graduate" nurse, the "practical" nurse, and the "nurse's aid." This is really what nurses want, although the fact is not generally understood, and only a body like the College can undertake the delicate task of segregating and labelling.

I have named two great and pressing tasks. There are a score of others not so great, but in their way equally important. And now I turn to the Executive, which consists almost exclusively of hospital matrons.

My very farthest thought is to utter a word, or even to harbour a thought, prejudicial to the matron. And, therefore, it is that I ask for the text. The matron necessarily represents the cream of the nursing profession. Like a naval officer she never asks her assistant to do anything that she could not do better herself. But the matron has her limitations. There are administrative duties of great importance connected with the College for which the average matron is wholly unfitted. And, moreover, what is more to the point, these women have not the necessary time at their disposal. They are literally submerged with their own duties. The College, in order to discharge its obligations with efficiency, ought to have the whole-time services of a large number of its ablest members for the next twelve months. There is much more to be done than in many a Government department or private firm, where the directors meet every day.

Those who love the nursing profession and the College will perhaps ponder on these things. I may be an alarmist. On the other hand I may not. In any case, I think nurses will do well to face their problems frankly, and not allow themselves to be shackled by tradition. We are in an age of rapid transition, and the time has arrived when abuses which had become intolerable must be swept out of existence. To accomplish this, and to leave only the best to survive, requires strength, courage, and sound judgment. It is to the College that the nurse looks for these attributes.

IERNE.

